# Bobble-head doll syndrome in an infant with an arachnoid cyst: a case report

**DOI:** 10.1186/s13256-022-03623-0

**Published:** 2022-10-28

**Authors:** Leen Jamel Doya, Hassan Kadri, Oday Jouni

**Affiliations:** 1 Department of Pediatrics, Faculty of Medicine, Tishreen University Hospital, Lattakia, Syria; 2 Department of Neurosurgery, Faculty of Medicine, Damascus University, Damascus, Syria

**Keywords:** Bobble-head doll syndrome, Suprasellar arachnoid cyst, Endoscopic cystoventriculostomy

## Abstract

**Background:**

Bobble-head doll syndrome is a rare and unique movement disorder most commonly affecting children younger than 5 years of age. It is characterized by continuous or episodic movement at the frequency of 2–3 Hz. The exact mechanism of bobble-head doll syndrome has not been elucidated. Endoscopic ventriculocisternostomy is the optimal treatment option. In a literature review, there were less than 75 cases of bobble-head doll syndrome with suprasellar arachnoid cyst.

**Case presentation:**

We report a case of a 1.5-year-old Asian-Syrian girl who presented with a history of excessive head nodding for 3 months that increased with walking, emotions, and stress; decreased during periods of concentration; and was absent during sleep. On physical examination, she was alert and normal, with no medical history. Laboratory assessment and ophthalmological examination were normal. Cranial magnetic resonance imaging demonstrated a well-defined thin-walled suprasellar arachnoid cyst measuring 3 × 5 × 7 cm that obstructed the foramina of Monro, with resulting hydrocephalus ventriculomegaly. The patient underwent endoscopic cystoventriculostomy and cystocisternostomy for the suprasellar arachnoid cyst. During the 6 months of follow-up, the head bobbing disappeared completely, and her growth was normal.

**Conclusion:**

Despite the rareness of bobble-head doll syndrome, it is considered an important condition that must be investigated early to detect the cause and treated promptly to avoid potential complications.

## Introduction

Bobble-head doll syndrome (BHDS) is a rare and unique movement disorder most commonly affecting children less than 5 years of age, and characterized by continuous or episodic forward and backward head nodding (yes–yes), or sometimes a side-to-side movement (no–no), at the frequency of 2–3 Hz. These movements disappear with volitional activity and are absent during sleep [1]. The first clinical case of the BHDS was noticed in 1966 by Benton in a child with hydrocephalus due to third ventricular cysts; fewer than 75 cases in children have been reported since then [2]. In a literature review published in 2018, the causes are commonly associated with a lesion in or around the third ventricle, causing it to dilate. The most common lesions are suprasellar arachnoid or third ventricular tumors, followed by aqueductal stenosis. Other causes include cysts of the cavum pellucidum and interpositum, developmental cerebellar disorders, communicating hydrocephalus, trapped fourth ventricle, and third ventricular choroid plexus papilloma [3]. The most common symptoms and signs, in addition to involuntary and repetitive movements, include developmental delay, macrocephaly, ataxia, optic disc pallor or atrophy, tremors, hyperreflexia, endocrine disorders (obesity, precocious puberty), headache, and vomiting [4]. The exact mechanisms underlying this movement disorder have not been elucidated. There are two main possible theories; the first hypothesis published by Russo and Klindt in 1974 is that BHDS is associated with dorsomedial compression caused by an abnormal flow of fluids towards the medial side of the thalamic nuclei. However, there are many opponents to this theory as not all expansions of the third ventricle lead to BHDS, and the symptom of typical extrapyramidal rigidity is absent [5]. The second hypothesis, by Wiese *et al.* in 1985, considers that an adopted motor automatism has been developed to decrease the pressure within the cyst [6]. The underlying causes of BHDS can be detected by computed axial tomography (CT) or magnetic resonance imaging (MRI) either without or with contrast, which is the best modality for the delineation of cerebrospinal fluid (CSF) pathways and soft tissue [4].

## Case presentation

A 1.5-year-old Asian-Syrian girl presented to the pediatric clinic with the chief complaints of gradual onset excessive head nodding (side-to-side movement) for 3 months. Movements increased with walking, emotions, and stress; decreased during periods of concentration; and were absent during sleep. There were no other complaints or headaches. There was no other significant history. Pregnancy and delivery were normal.

On physical examination, the child was alert, with normal cognitive function. Neurological examination was normal. She had normal growth (weight 8 kg, length 72 cm, head circumference 44.5 cm).

Initial laboratory assessment including complete blood count (CBC), hepatic and renal function, and endocrine function tests were normal.

**Fig. 1:**
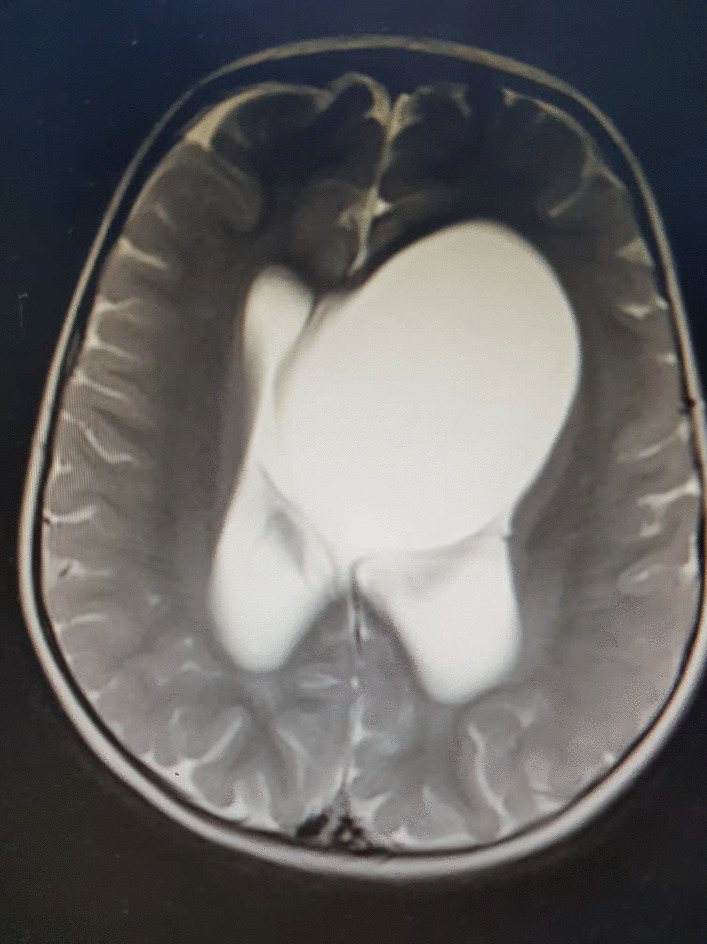
Cranial MRI imaging demonstrated a large left-hemispheric cystic process with a midline shift, well-defined thin-walled suprasellar arachnoid cyst measuring 3 × 5 × 7 cm causing hydrocephalus ventriculomegaly

An ophthalmological examination revealed normal eye movements with no papilledema. Cranial MRI imaging demonstrated a large left-hemispheric cystic process with a midline shift, well-defined thin-walled suprasellar arachnoid cyst measuring 3 × 5 × 7 cm that obstructed the foramina of Monro, with resulting hydrocephalus ventriculomegaly (Fig. 1). Based on the cranial MRI and symptoms, a diagnosis of a suprasellar arachnoid cyst with BHDS was made.

**Fig. 2:**
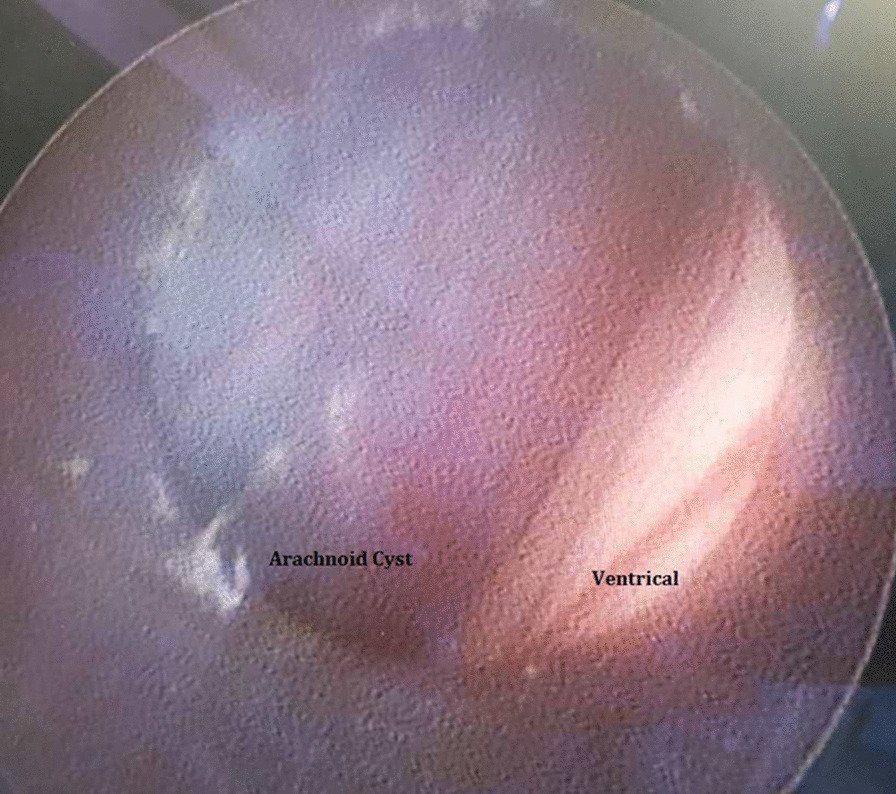
Endoscopic imaging identified an arachnoid cyst with ventricle

The patient underwent endoscopic cystoventriculostomy and cystocisternostomy for the suprasellar arachnoid cyst (Fig. 2). The surgery was performed through a small para coronal burr hole on the right side of the skull. Insertion of the endoscopic trocar identified clear CSF that appeared to be under pressure. Very quickly, the cyst jumped towards our lens due to the high intracystic pressure. This high pressure seemed to have been compensated by the back pressure of the CSF. The fenestration was done very quickly. The cyst fluid seemed very clear and under pressure. With the bipolar probe, we removed a large part of the wall. Many other small fenestration holes were made in the anterior direction and at the bottom. At the end of the procedure, we achieved normal pulsation of the ventricle, with a good flow of fluid around the remaining membrane.

At follow-up 6 weeks after the procedure, she had a reduction in both the frequency and intensity of head movements. During the 6 months of follow-up, the head bobbing disappeared completely, and her growth was normal.

## Discussion and conclusion

The exact incidence of arachnoid cysts is unknown because many may be asymptomatic throughout life. However, symptoms can occur when the cysts enlarge or hemorrhage. Recorded cases account for approximately 1% of all intracranial lesions, and 9% of them are suprasellar. The association between BHDS and suprasellar arachnoid cysts is extremely rare, but has been described in the medical literature [7]. Almost all the cases in the literature review were symptomatic of head movements, as in Table 1; this may be either due to the large size of the cyst or the delayed diagnosis.

**Table 1:** Cases of BHDS with arachnoid cyst from the literature review

The author/date	The patient (age, sex)	Manifestation	Diagnosis	Treatment
Roshan 2020	8-year-old M	Involuntary bobbling head movements.	MRI of the brain: an extensive suprasellar cyst resulting in obstructive hydrocephalus	Endoscopic ventriculocystocisternostomy
Saracoglu 2019	9‐year-old F	Involuntary abnormal head movements.	MRI: suprasellar anterior third ventricular arachnoid cyst and obstructive hydrocephalus.	Cysto-ventriculocisternostomy by endoscopic fenestration.
Renne 2018	14-year-old M.	Involuntary abnormal head movements with atriventricular hydrocephalus	MRI: suprasellar anterior third ventricular arachnoid cyst and obstructive hydrocephalus.	Endoscopic third ventriculostomy
	7.5-year-old F	BHDS with preexisting endocrinopathy and autism spectrum disorder at the age of 12 years	MRI: suprasellar arachnoid cyst	Endoscopic fenestration
Ramesh 2015	10-month-old F	Two episodes of generalized tonic-clonic seizures Involuntary bobbling of the head for a month	MRI: intensity cystic lesion in prepontine, interpeduncular, suprasellar anterior third ventricle with moderate dilatation of third and both lateral ventricles	Right frontal burr hole, endoscopic partial excision and marsupialization of cyst, third ventriculostomy, and septostomy
	4-year-old F	Involuntary nodding of the head back Ataxia on walking since 1 year	CT scan: dilatation of lateral ventricles and third ventricles MRI: large suprasellar arachnoid cyst in the third ventricle with compression of the fornix, corpus callosum with enlarged lateral and third ventricles.	Frontal burr hole, endoscope marsupialization of the cyst, cystoventriculostomy, and third ventriculostomy
	8-year-old F	Fever on and off for 2 months Difficulty in walking Abnormal head movements for 1 month One episode of generalized tonic-clonic seizures	CT and MRI brain: large third ventricular arachnoid cyst with mass effect over the brain stem, and obstructive hydrocephalus	Frontal burr hole, endoscopic cystoventriculostomy, third ventriculostomy and reservoir placement
Reddy 2014	9-year-old M	Involuntary movements of head as excessive head nodding since 1 year. Clinical examination: conscious and coherent fundus, temporal pallor, more on the right side	CT scan and MRI of the brain: cystic lesion arising from the suprasellar region extending upwards causing compression of the third ventricle leading to obstructive hydrocephalus at the level of the third ventricle.	Ventriculocystostomy endoscopically
Muthusubramanian 2006	5 year-old M	Intermittent head nodding of 2 years duration associated with tremors of the hands since 1.5 years.	CT and MRI scans of the brain revealed a suprasellar arachnoid cyst with obstructive hydrocephalus	Endoscopic ventriculocystocisternostomy
Eveline 2005	4-year-old M	BHDS	CT and MRI scans: suprasellar arachnoid cysts	An endoscopic cystoventriculostomy was performed
Fioravanti 2004	1-year-old F	Persistent nocturnal tearing abnormal head movements.	CT and MRI: a bulky arachnoid cyst of the suprasellar region that occupied the third ventricle and obstructed the foramina of Monro, causing biventricular hydrocephalus	Ventriculocystocisternostomy was endoscopically
	9-year-old M	Early puberty for the previous 9 months Behavioral problems and enuresis. Clinical examination: clumsy gait and abnormal head trunk movements.	CT and MRI: arachnoid cyst of the suprasellar region that occupied the third ventricle and obstructed the foramina of Monro, causing notable biventricular hydrocephalus	Ventriculocystostomy was endoscopically
Desai 2003	Three cases with a mean of 3.3 years old	The duration of complaints ranged from 4 to 6 months, a clinical complaint was BHDS	CT and MRI scans: suprasellar arachnoid cysts	Third patient: the massive cyst was exposed through a transcallosal route. First and second patients: a translaminar approach was adopted to expose the cyst.

*F* Female,* M* Male,* Yr* Year,* CT* A computerized tomography,* MRI* Magnetic resonance imaging,* BHDS* Bobble-head doll syndrome

Our patient had no significant history, with normal examination excluding excessive side-to-side head nodding. A diagnosis of a suprasellar arachnoid cyst with BHDS was made based on the cranial MRI and symptoms.

The treatment of BHDS is based on the treatment of the primary lesion, and is usually surgical. It was treated before establishing neuroendoscopic techniques, with open marsupialization or permanent ventriculoperitoneal or cystoperitoneal drainage [8]. Nowadays neuroendoscopic interventions with ventriculocystocisternostomy have gained popularity as a favorable therapeutic option for arachnoid cysts owing to successful results, while being less invasive [9].

In summary, we report our successful experience of neuroendoscopic interventions with ventriculocystocisternostomy for the treatment of arachnoid cysts in a newborn with BHDS. Despite the rareness of the BHDS, it is considered an important condition that must be investigated early to detect the cause and treated promptly to avoid potential complications.

## Data Availability

All data generated or analyzed during this study are included in this published article.
